# Supplementary material for: Inhibition of DNA2 nuclease as a therapeutic strategy targeting replication stress in cancer cells
Source: Oncogenesis. 2017 Apr 17;6(4):e319–. doi: 10.1038/oncsis.2017.15 (PMC5520492; doi:10.1038/oncsis.2017.15)
Supplement: Supplementary Figures [file oncsis201715x1.ppt]

## Slide 1
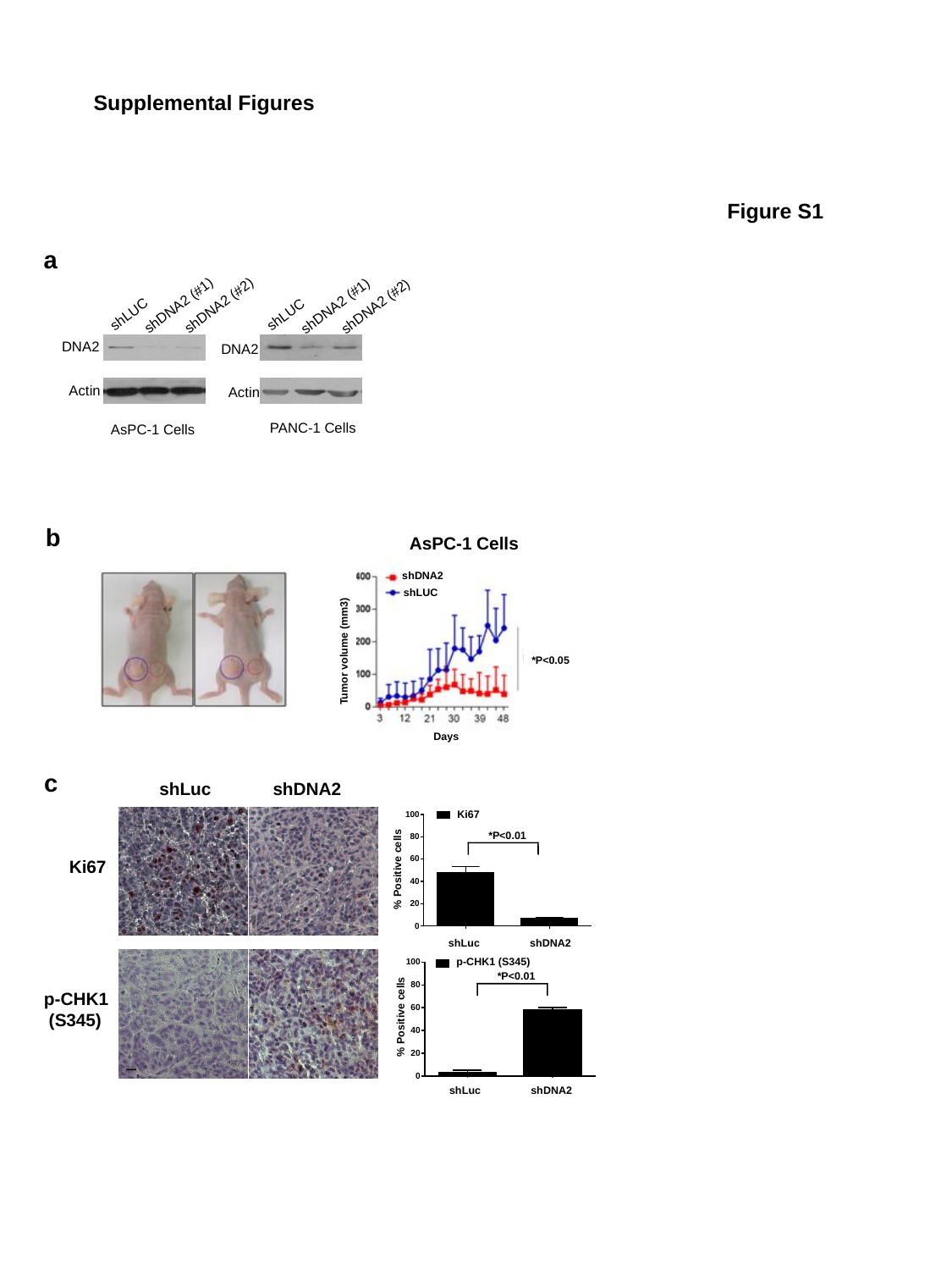

Supplemental Figures
Figure S1
a
shDNA2 (#1)
shDNA2 (#2)
shDNA2 (#1)
shDNA2 (#2)
shLUC
shLUC
DNA2
DNA2
Actin
Actin
PANC-1 Cells
AsPC-1 Cells
b
AsPC-1 Cells
shDNA2
shLUC
Tumor volume (mm3)
*P<0.05
Days
c
shLuc
shDNA2
Ki67
*P<0.01
Ki67
% Positive cells
shLuc
shDNA2
p-CHK1 (S345)
*P<0.01
p-CHK1
 (S345)
% Positive cells
shLuc
shDNA2

## Slide 2
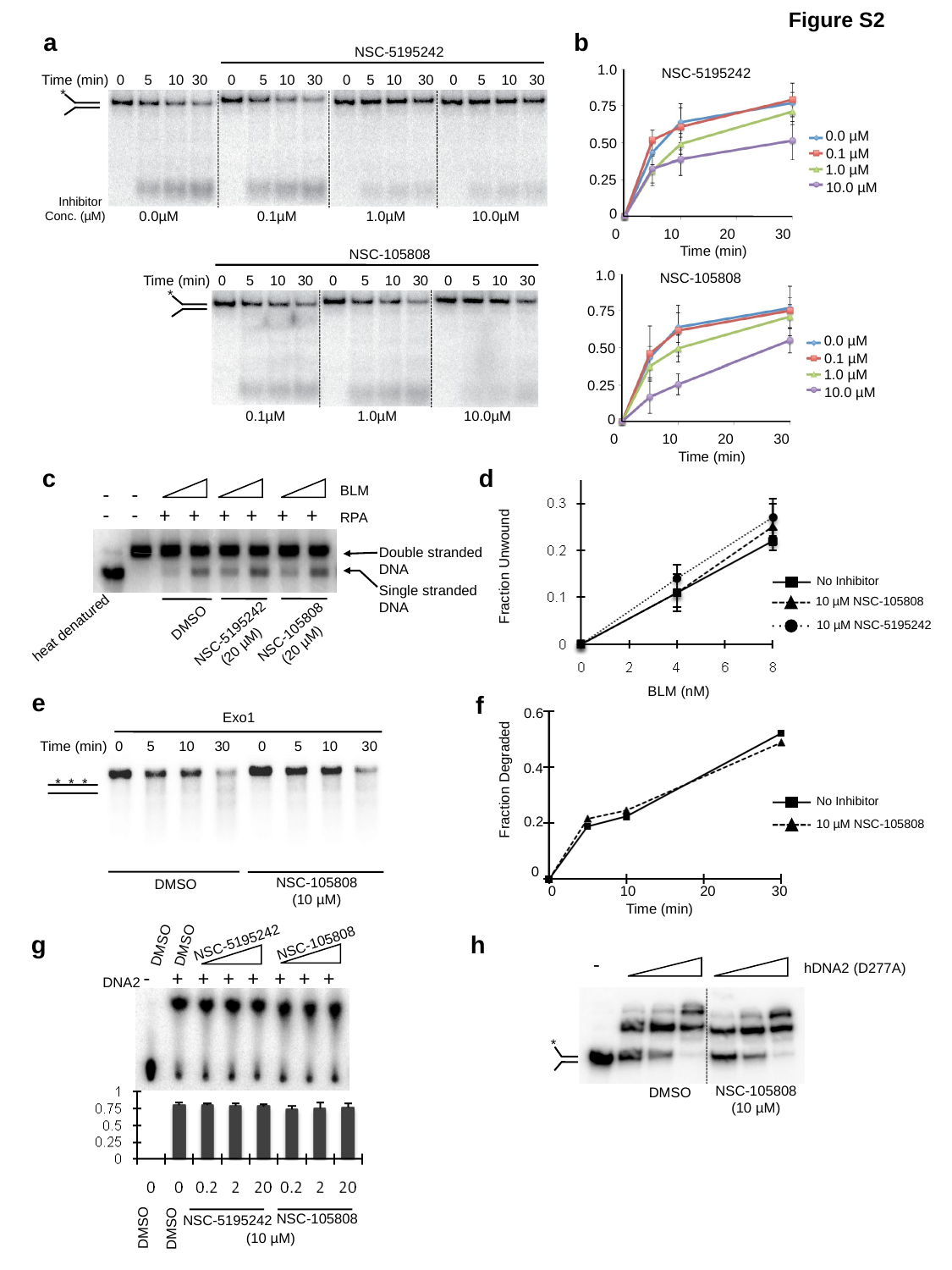

Figure S2
a
b
NSC-5195242
1.0
NSC-5195242
0.75
0.0 µM
0.50
0.1 µM
1.0 µM
0.25
10.0 µM
0
0 10 20 30
Time (min)
Time (min) 0 5 10 30 0 5 10 30 0 5 10 30 0 5 10 30
*
Inhibitor
Conc. (µM)
10.0µM
0.0µM
0.1µM
1.0µM
NSC-105808
Time (min) 0 5 10 30 0 5 10 30 0 5 10 30
*
10.0µM
0.1µM
1.0µM
1.0
NSC-105808
0.75
0.0 µM
0.50
0.1 µM
1.0 µM
0.25
10.0 µM
0
0 10 20 30
Time (min)
c
d
-
-
BLM
-
-
+
+
+
+
+
+
RPA
Double stranded DNA
Fraction Unwound
No Inhibitor
10 µM NSC-105808
10 µM NSC-5195242
Single stranded DNA
DMSO
heat denatured
NSC-105808 (20 µM)
NSC-5195242
(20 µM)
BLM (nM)
e
Exo1
Time (min) 0 5 10 30 0 5 10 30
* * *
NSC-105808
(10 µM)
DMSO
f
0.6
0.4
Fraction Degraded
No Inhibitor
10 µM NSC-105808
0.2
0
0 10 20 30
Time (min)
DMSO
DMSO
NSC-5195242
NSC-105808
-
+
+
+
+
+
+
+
DNA2
DMSO
DMSO
NSC-105808
NSC-5195242
g
h
-
hDNA2 (D277A)
*
NSC-105808
(10 µM)
DMSO
(10 µM)

## Slide 3
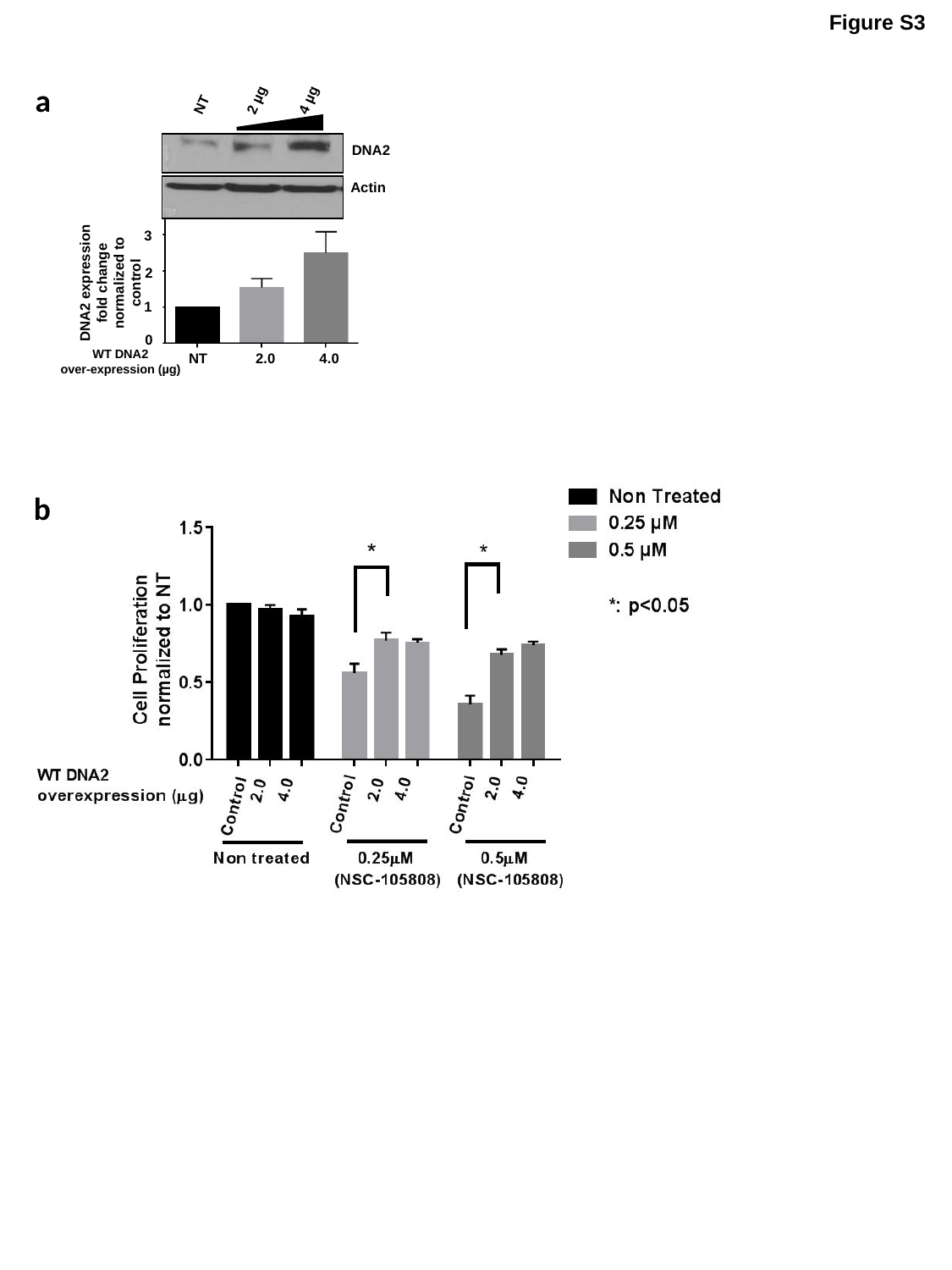

Figure S3
4 µg
NT
2 µg
DNA2
4
3
DNA2 expression fold change normalized to control
2
1
0
WT DNA2
over-expression (µg)
NT 2.0 4.0
Actin
a
b

## Slide 4
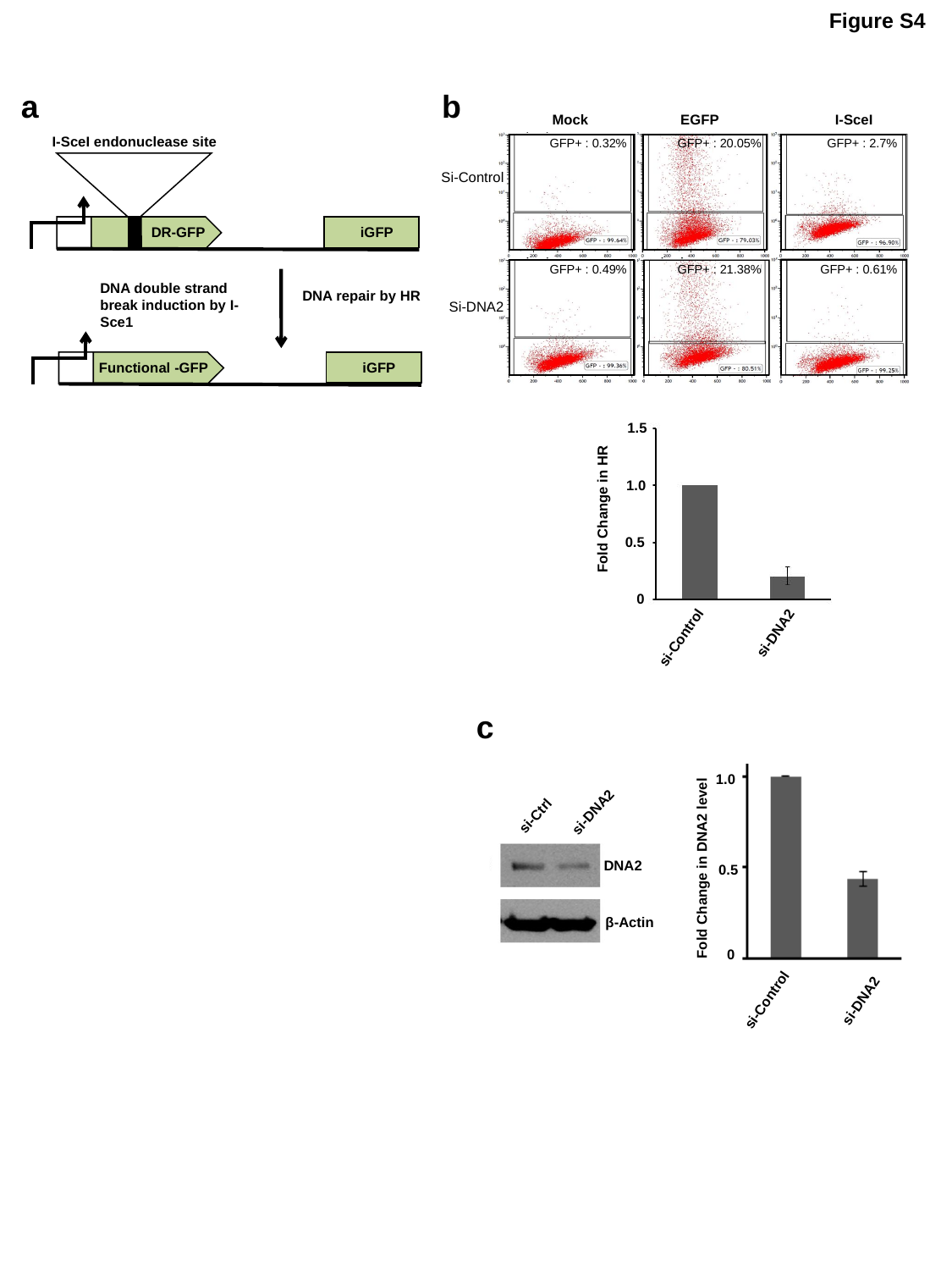

Figure S4
a
b
 Mock EGFP I-SceI
I-SceI endonuclease site
GFP+ : 0.32%
GFP+ : 20.05%
GFP+ : 2.7%
Si-Control
DR-GFP
iGFP
GFP+ : 0.49%
GFP+ : 21.38%
GFP+ : 0.61%
DNA double strand break induction by I-Sce1
DNA repair by HR
Si-DNA2
Functional -GFP
iGFP
1.5
1.0
Fold Change in HR
0.5
0
si-DNA2
si-Control
c
1.0
si-DNA2
si-Control
Fold Change in DNA2 level
0.5
0
si-DNA2
si-Ctrl
DNA2
β-Actin

## Slide 5
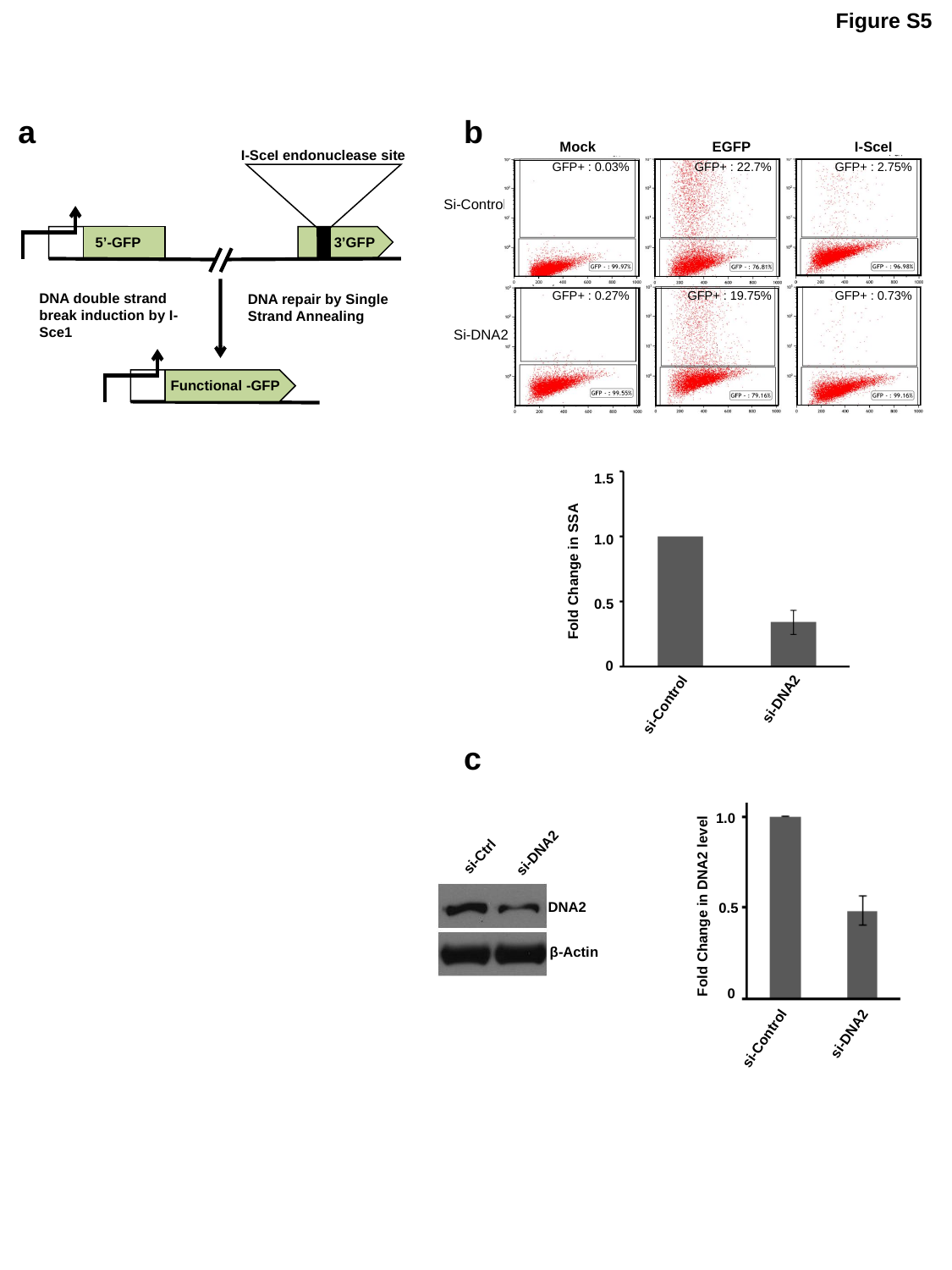

Figure S5
a
b
 Mock EGFP I-SceI
GFP+ : 0.03%
GFP+ : 22.7%
GFP+ : 2.75%
GFP+ : 0.27%
GFP+ : 19.75%
GFP+ : 0.73%
Si-DNA2
I-SceI endonuclease site
Si-Control
5’-GFP
3’GFP
DNA double strand break induction by I-Sce1
DNA repair by Single Strand Annealing
Functional -GFP
1.5
1.0
Fold Change in SSA
0.5
0
si-DNA2
si-Control
c
1.0
Fold Change in DNA2 level
0.5
0
si-DNA2
si-Control
si-DNA2
si-Ctrl
DNA2
β-Actin

## Slide 6
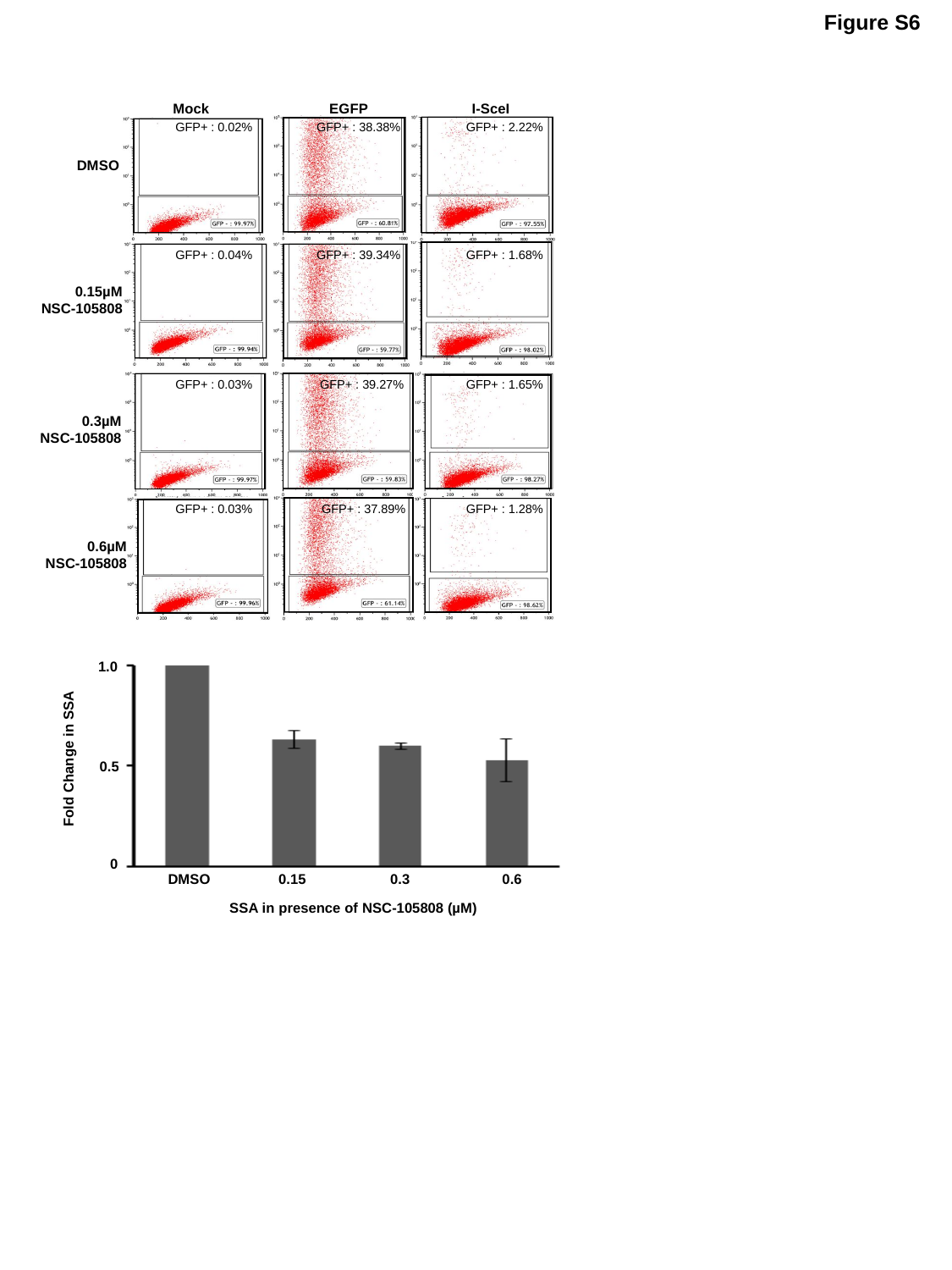

Figure S6
 Mock EGFP I-SceI
DMSO
0.15µM
NSC-105808
0.3µM
NSC-105808
0.6µM
NSC-105808
GFP+ : 0.02%
GFP+ : 38.38%
GFP+ : 2.22%
GFP+ : 0.04%
GFP+ : 39.34%
GFP+ : 1.68%
GFP+ : 0.03%
GFP+ : 39.27%
GFP+ : 1.65%
GFP+ : 0.03%
GFP+ : 37.89%
GFP+ : 1.28%
1.0
Fold Change in SSA
0.5
0
DMSO 0.15 0.3 0.6
SSA in presence of NSC-105808 (µM)

## Slide 7
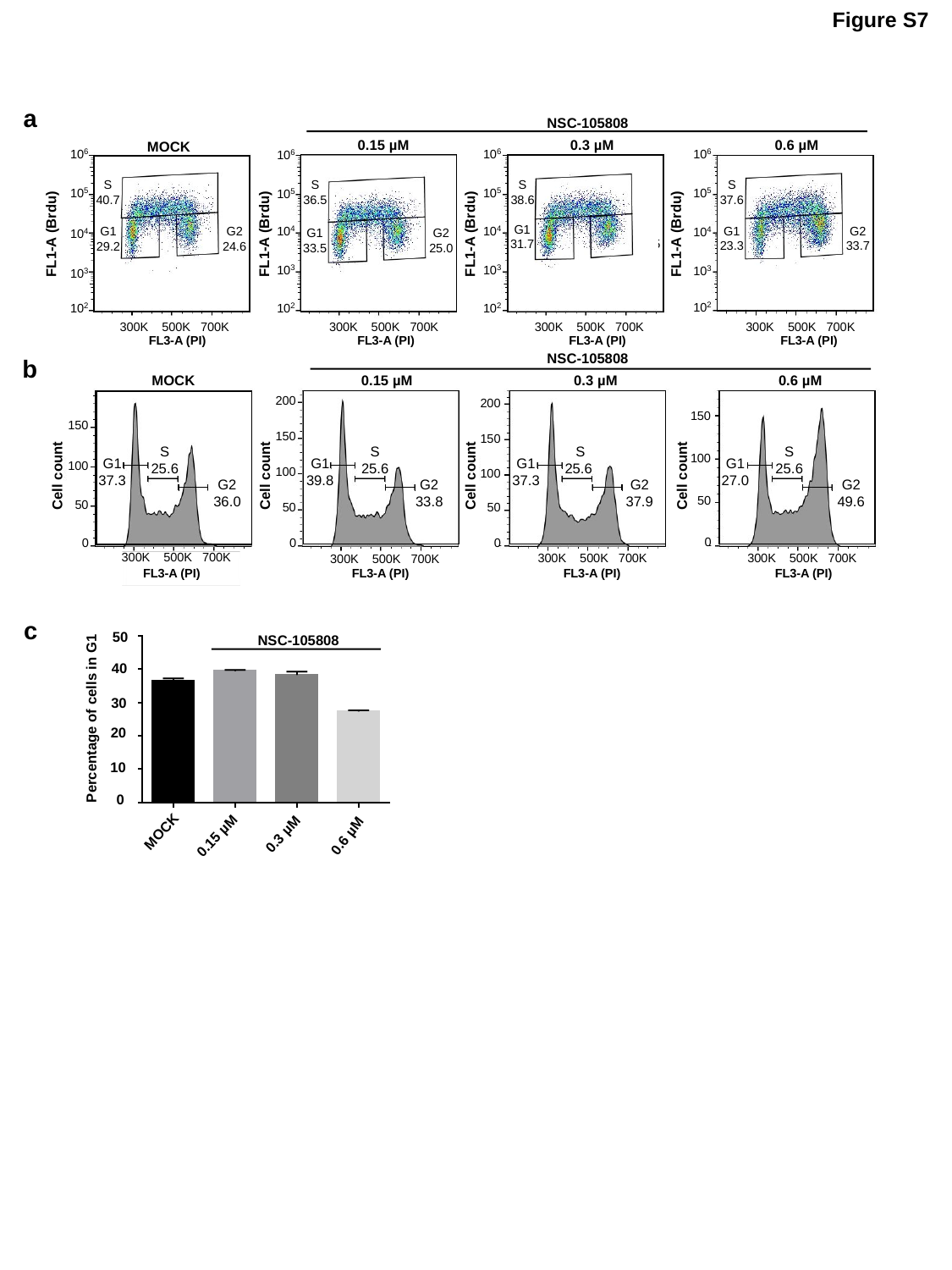

Figure S7
a
MOCK
106
S
40.7
105
104
G1
29.2
G2
24.6
FL1-A (Brdu)
103
102
300K 500K 700K
FL3-A (PI)
NSC-105808
0.3 µM
106
S
38.6
105
G1
31.7
104
FL1-A (Brdu)
103
102
300K 500K 700K
FL3-A (PI)
0.15 µM
106
S
36.5
105
104
G1
33.5
G2
25.0
FL1-A (Brdu)
103
102
300K 500K 700K
FL3-A (PI)
0.6 µM
106
S
37.6
105
104
G1
23.3
G2
33.7
103
102
300K 500K 700K
FL3-A (PI)
G2
25.5
FL1-A (Brdu)
NSC-105808
0.3 µM
S
25.6
G1
37.3
G2
37.9
b
MOCK
S
25.6
G1
37.3
G2
36.0
0.15 µM
S
25.6
G1
39.8
G2
33.8
0.6 µM
S
25.6
G1
27.0
G2
49.6
200
150
100
50
0
200
150
150
100
50
0
150
100
100
Cell count
Cell count
Cell count
Cell count
50
50
0
0
300K 500K 700K
300K 500K 700K
300K 500K 700K
300K 500K 700K
FL3-A (PI)
FL3-A (PI)
FL3-A (PI)
FL3-A (PI)
c
50
NSC-105808
40
30
Percentage of cells in G1
20
10
0
MOCK
0.3 µM
0.6 µM
0.15 µM

## Slide 8
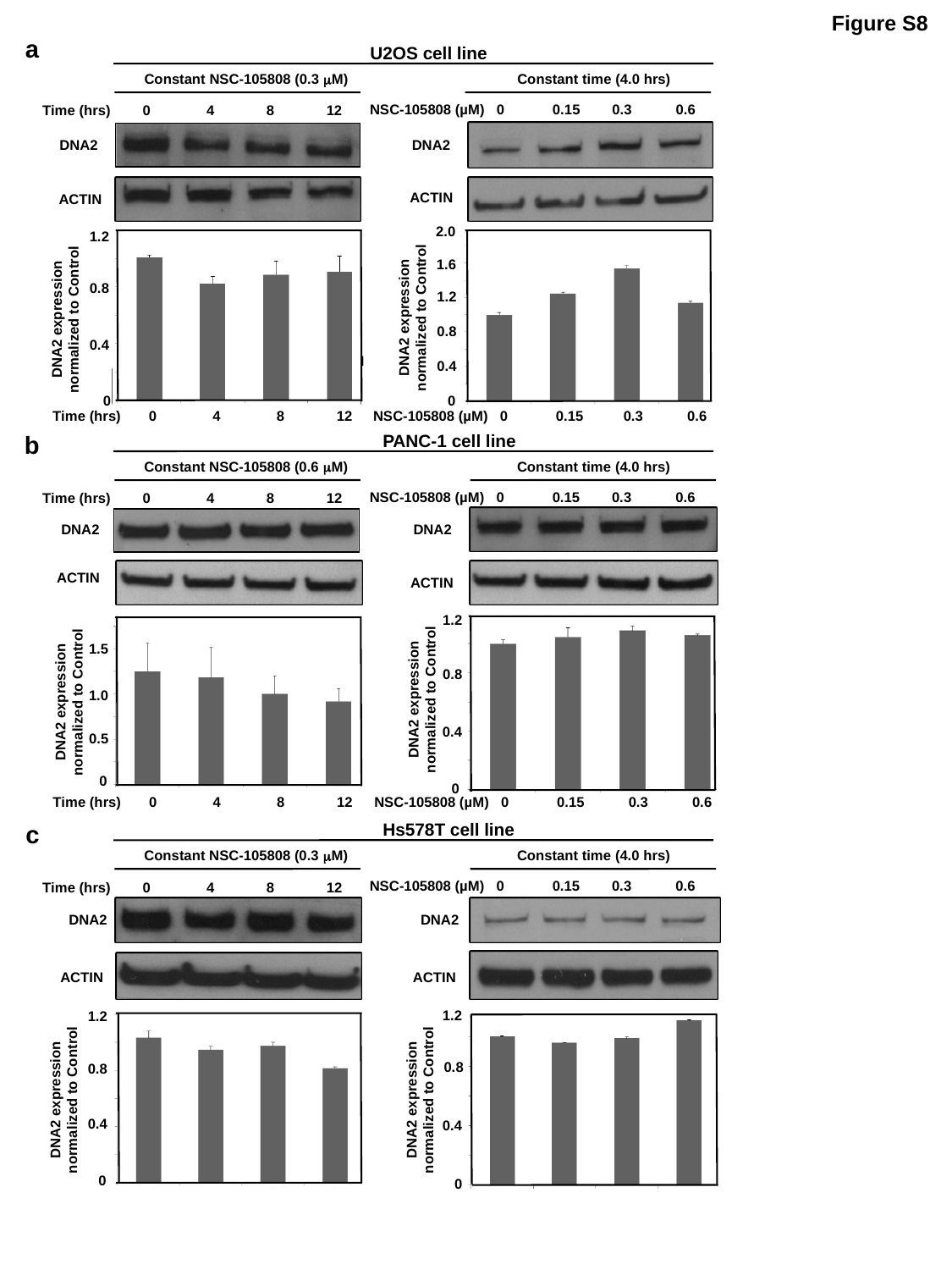

Figure S8
a
U2OS cell line
Constant NSC-105808 (0.3 μM)
Constant time (4.0 hrs)
NSC-105808 (µM) 0 0.15 0.3 0.6
Time (hrs) 0 4 8 12
DNA2
DNA2
ACTIN
ACTIN
2.0
1.2
1.6
0.8
1.2
DNA2 expression normalized to Control
DNA2 expression normalized to Control
0.8
0.4
0 0.15 0.3 0.6 μM
0.4
0
0
Time (hrs) 0 4 8 12
NSC-105808 (µM) 0 0.15 0.3 0.6
b
PANC-1 cell line
Constant NSC-105808 (0.6 μM)
Constant time (4.0 hrs)
NSC-105808 (µM) 0 0.15 0.3 0.6
Time (hrs) 0 4 8 12
DNA2
DNA2
ACTIN
ACTIN
1.2
1.5
0.8
DNA2 expression normalized to Control
DNA2 expression normalized to Control
1.0
0.4
0.5
0
0
Time (hrs) 0 4 8 12
NSC-105808 (µM) 0 0.15 0.3 0.6
Hs578T cell line
Constant NSC-105808 (0.3 μM)
Constant time (4.0 hrs)
NSC-105808 (µM) 0 0.15 0.3 0.6
Time (hrs) 0 4 8 12
c
DNA2
DNA2
ACTIN
ACTIN
1.2
1.2
0.8
0.8
DNA2 expression normalized to Control
DNA2 expression normalized to Control
0.4
0.4
0
0

## Slide 9
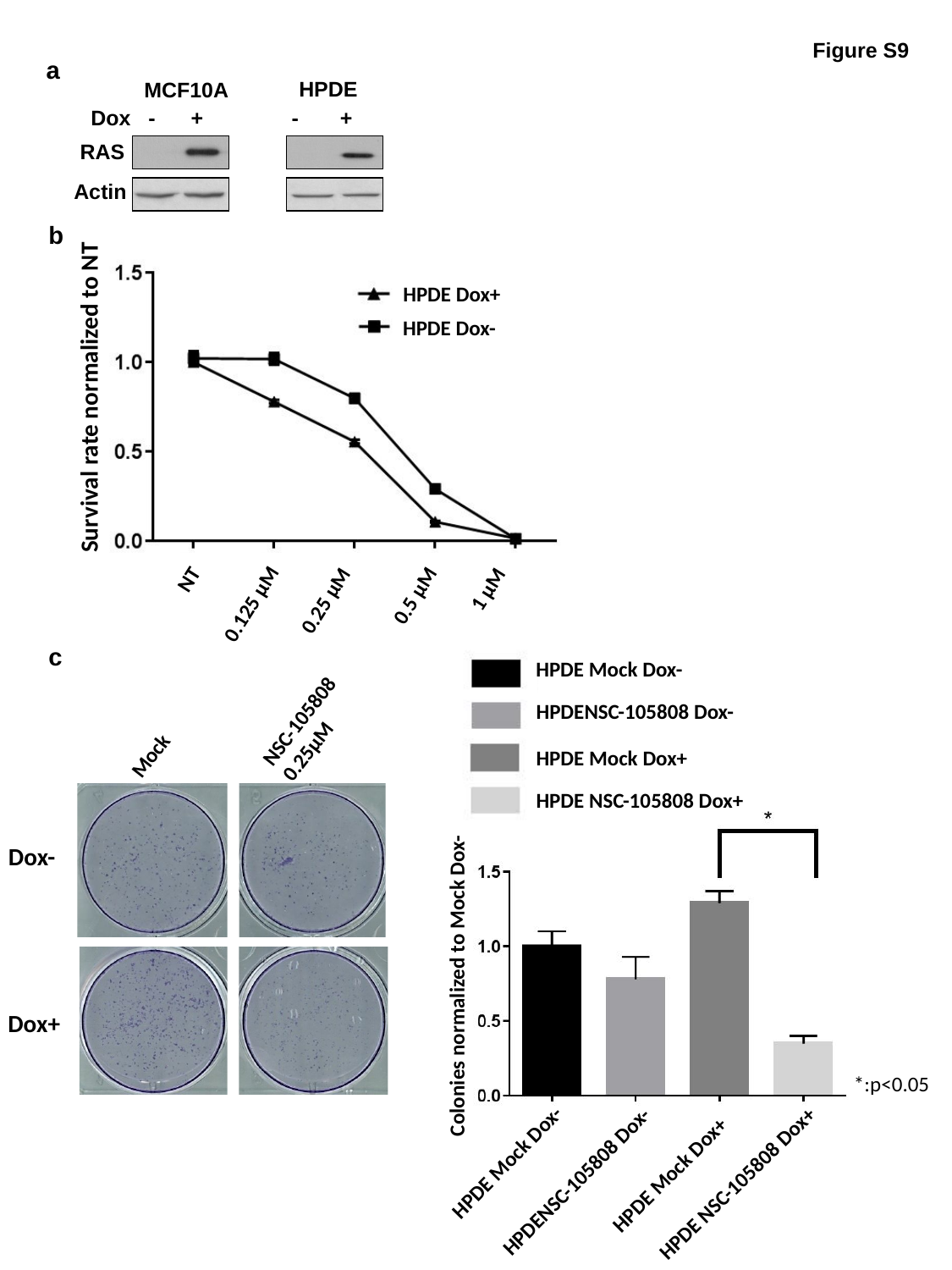

Figure S9
a
HPDE
MCF10A
Dox - + - +
RAS
Actin
b
HPDE Dox+
HPDE Dox-
Survival rate normalized to NT
1 µM
NT
0.5 µM
0.25 µM
0.125 µM
c
HPDE Mock Dox-
NSC-105808
0.25µM
Mock
HPDENSC-105808 Dox-
HPDE Mock Dox+
HPDE NSC-105808 Dox+
Dox-
Colonies normalized to Mock Dox-
Dox+
*:p<0.05
HPDE Mock Dox-
HPDENSC-105808 Dox-
HPDE NSC-105808 Dox+
HPDE Mock Dox+
