# Supplementary material for: Inhibition of DNA2 nuclease as a therapeutic strategy targeting replication stress in cancer cells
Source: Oncogenesis. 2017 Apr 17;6(4):e319–. doi: 10.1038/oncsis.2017.15 (PMC5520492; doi:10.1038/oncsis.2017.15)
Supplement: Supplementary Tables [file oncsis201715x2.docx]

**Supplemental Material**

**Supplemental Tables**

**Table S1.** Chemical libraries used in the screen for DNA2 inhibitors.

| **Library name** | **Number of compounds** |
| --- | --- |
| Microsource Spectrum Collection of US Drug compounds and natural product set | 147 |
| ChemBridge DIVERSet-1 | 30080 |
| Prestwick Chemical library | 1120 |
| NIH Clinical collection | 471 |
| NCI mechanistic set | 2715 |
| Maybridge collection | 14400 |

**Table S2.** Compounds that inhibit yDna2 but not T5 nuclease identified in the primary screen.

| **Library Name** | **Compound Name** | **IC50 from Screening** | |
| --- | --- | --- | --- |
|  |  | **In Vitro (µM)** | **Cell Based (µM)** |
| **Custom Clinical** | BIBW2992 | 7.2 | 1.2 |
| **NCI mechanistic set** | 57103 | 21 | 0.5 |
|  | 651084 | 1.7 | 5.7 |
|  | 123418 | 10.3 | n.d. |
|  | 45383 | 2.5 | n.d. (cytotoxic) |
|  | 83950 | 1.6 | n.d. (cytotoxic) |
|  | 111041 | 1 | n.d. |
|  | 105808 | 1.5 | 2.7 |
|  | 45383 | 5.9 | n.d. (cytotoxic) |
|  | 337766 | 3.5 | 0.3 |
|  | 659999 | n.d. | n.d. |
|  | 253995 | 2.3 | n.d. |
| **ChemBridge DIVERSet-1** | 7734235 | n.d. | n.d. |
|  | 7749960 | 17.1 | n.d. |
|  | 7804572 | 5.5 | 8.3 |
|  | 7814291 | 6.6 | 6.8 |
|  | 5132326 | n.d. | n.d. |
|  | 5193784 | n.d. | n.d. |
|  | 5194440 | 17.1 | 6.4 |
|  | 5194421 | 19.5 | 5.2 |
|  | 5194408 | 18.2 | 7.5 |
|  | 5195242 | 2.6 | 1.0 |
|  | 5226755 | 18.2 | 5.7 |
|  | 5226973 | n.d. | 4 |
|  | 5276201 | 17.1 | 5.2 |
|  | 5278045 | 23.8 | 7.5 |
|  | 5314531 | 22.3 | 5.2 |
|  | 5318845 | 2.6 | 9.1 |
|  | 5319866 | 17.5 | 6.9 |
|  | 5321683 | 4.2 | 2.4 |
|  | 5320337 | 11.5 | 3.6 |
|  | 5320788 | 12.3 | 2.7 |
|  | 5322666 | 20.8 | 2.9 |
| **Maybridge collection** | JA00082SC | 3 | n.d. |
|  | BTB02226SC | n.d. | 8.3 |
|  | CD04412SC | 12.3 | n.d. |
|  | BTB02990SC | 10.7 | 6.8 |
|  | JFD01947SC | 2.3 | 2.7 |
|  | KM06839SC | 13 | 1.3 |

**Table S3.** Compounds that inhibit both yDna2 and T5 nuclease.

| **Library Name** | **Compound Name** | **% Inhibition** | | **Molecular Weight** |
| --- | --- | --- | --- | --- |
|  |  | **yDna2** | **T5 nuclease** |  |
| Custom Clinical | NSC23766 | 100 | 94.7 | 458.04 |
|  | BI2536 | 55.3 | 77.7 | 521.65436 |
|  | (Dovitinib) CHIR258 | 54 | 60.2 | 392.4 |
| Prestwick Chemical Library | Propidium Iodide | 100 | 100 | 668.39 |
|  | Deqalinium dichloride | 100 | 100 | 527.5714 |
|  | Mitoxantrone dihydrochloride | 100 | 100 | 517.4 |
|  | Doxorubicin hydrochloride | 100 | 100 | 579.98 |
|  | Daunorubicin hydrochloride | 100 | 98.1 | 563.98 |
|  | Chicago Sky blue 6B | 69 | 100 | 992.8 |
|  | Quinacrine dihydrochloride | 67.8 | 55.4 | 472.88 |
|  | Alexidine dihydrochloride | 63.5 | 100 | 581.71 |
| NCI mechanistic set | NSC309401 | 100 | 100 | 377 |
|  | NSC33353 | 100 | 100 | 338 |
|  | NSC311153 | 100 | 100 | 434 |
|  | NSC67436 | 100 | 100 | 487 |
|  | NSC60339 | 100 | 100 | 487 |
|  | NSC70929 | 100 | 100 | 747 |
|  | NSC260610 | 100 | 100 | 531 |
|  | NSC317605 | 100 | 99.2 | 368 |
|  | NSC143491 | 100 | 97.9 | 579 |
|  | NSC36758 | 100 | 83.9 | 306 |
|  | NSC363998 | 99 | 74.5 | 513 |
|  | NSC260594 | 94.8 | 100 | 505 |
|  | NSC622124 | 93.4 | 96.1 | 3015 |
|  | NSC10010 | 90.3 | 93.7 | 574 |
|  | NSC219734 | 85 | 100 | 499 |
|  | NSC177365 | 83.3 | 100 | 566 |
|  | NSC146771 | 76.5 | 100 | 476.49 |
|  | NSC273829 | 74.4 | 56.5 | 443 |
|  | NSC343256 | 71 | 72 | 478 |
|  | NSC166454 | 55.4 | 88.9 | 528 |
|  | NSC344494 | 51.4 | 97.9 | 175 |
| ChemBridge DIVERSet-1 | 5683601 | 69.8 | 81 | 418 |
|  | 5228301 | 65.8 | 88.2 | 311 |
|  | 5917278 | 62.4 | 100 | 304 |
|  | 5308753 | 62 | 58.6 | 384 |
|  | 5309857 | 59.9 | 77.3 | 311 |
| Maybridge collection | MB00046 | 64.1 | 100 | 304.355 |
|  | JFD03185 | 62.4 | 71.8 | 278.262 |
|  | JFD00884 | 61.7 | 49 | 379.66 |
|  | BTB10320 | 53.6 | 55.4 | 452.33 |
|  | JFD00257 | 52 | 95.9 | 279.32 |
