# Supplementary material for: Inhibition of DNA2 nuclease as a therapeutic strategy targeting replication stress in cancer cells
Source: Oncogenesis. 2017 Apr 17;6(4):e319–. doi: 10.1038/oncsis.2017.15 (PMC5520492; doi:10.1038/oncsis.2017.15)
Supplement: Supplementary Figure Legends [file oncsis201715x3.docx]

**Supplemental Material**

**Supplemental Figure Legends**

**Figure S1.** DNA2 knockdown in pancreatic cancer cell lines. (**a**) Western blot analysis of DNA2 knockdown efficiency in indicated cell lines. (**b**) DNA2 knockdown inhibits pancreatic cancer xenograft tumor growth. (Left) representative images of AsPC-1 xenografts with control and DNA2 knockdown cells. (Right) control and DNA2 knockdown cells were inoculated subcutaneously in athymic nu/nu mice. Average tumr volume was plotted against days of tumor growth (n=5 for each group, *p<0.05). (**c**) Representative examples of the immunohistochemistry analyses of xenograft tumors with anti-Ki67, a proliferative marker and anti-pCHK1 (S345), a DNA damage response marker. Scale bar, 50µM. Quantification of anti-Ki67 and anti-pCHK1 (S345)-positive cells of three individual tumors. *p<0.01.

**Figure S2.** NSC-105808 and NSC-5195242 affect DNA flap processing but not DNA binding by DNA2 or DNA unwinding by BLM. (**a**) Analysis of 5’ flap processing by hDNA2 in presence of the compounds at the indicated concentration. DNA2 (2.5 nM) was incubated with the Y DNA substrate (5 nM ends) and the indicated concentration of inhibitor. (**b**) Plots showing quantitation of the experiments shown in **a**. Plotted are the average data from three independent experiments with the error bars representing one standard deviation. (**c**) Analysis of dsDNA unwinding by BLM. BLM (4, 8 and 16 nM), RPA (100 nM) and internally radiolabelled dsDNA (0.5 nM ends) was incubated for 20 min at 37^º^C in presence of either DMSO or NSC-105808 (10 µM) and the products were resolved on a 1% agarose gel. (**d**) Plots showing quantitation of the experiments shown in **c**. Plotted are the average data from three independent experiments with the error bars representing one standard deviation. (**e**) Analysis of the degradation of a 2 kb internally labeled dsDNA (0.5 nM ends) by EXO1 (5 nM) in presence of NSC-105808 (10 µM). (**f**) Plot showing quantitation of the experiment in **e**. (**g**) NSC-105808 and NSC-5195242 do not affect the ATPase activity of hDNA2. 100 nM hDNA2 was incubated with γ^32^P-ATP in the presence of oligonucleotide ssDNA (200 nM) for 20 minutes at 37^º^C in presence of either DMSO or inhibitor (10 µM) and the products were resolved on a TLC plate. (**h**) Analysis of hDNA2 binding to DNA. Electrophoretic mobility shift assay was carried out with nuclease dead hDNA2-D277A. hDNA2-D277A (5, 10 and 20 nM) was incubated with 2.5 nM Y shaped DNA in presence of either DMSO or NSC-105808 (10 µM) for 10 min at 25^o^C and the reaction mixtures were resolved in a 7.5% native polyacrylamide gel.

**Figure S3.** DNA2 overexpression reduces sensitivity of U2OS cells to NSC-105808. (**a**) Western blot analysis of DNA2 from U2OS cells transfected with DNA2. Quantitation of Western blot is shown, with error bars representing standard deviation (n=3). (**b**) Proliferation rates of U2OS cells with the endogenous level of DNA2 (control) or overexpressing DNA2 and either non treated or treated with 0.25 µM and 0.5 µM of NSC-105808 for 48 hours. The average data from three independent experiments are shown, with the error bars representing standard deviation.

**Figure S4.** DNA2 knockdown reduces HR efficiency. (**a**) Schematic of the gene conversion assay involving the use of the DR-GFP reporter in the U2OS cell line. (**b**) Cells were transfected with either control siRNA or DNA2 siRNA for 24 hrs, followed by EGFP or I-SceI transfection for an additional 48 hrs. Cells were collected and analyzed by flow cytometry (top). Quantitation of the fold change in HR is shown below. (**c**) Western blot analysis of DNA2 knockdown. The average data from three independent experiments are shown, with the error bars representing standard deviation (n=3).

**Figure S5.** DNA2 knockdown reduces DSB repair by single strand annealing (SSA). (**a**) Schematic of the SSA assay in U2OS cell line. (**b**) Cells were transfected with either control or DNA2 siRNA for 24 hrs, followed by EGFP/I-SceI transfection for 48 hrs. Cells were collected and analyzed by flow cytometry (top) and quantitation of the fold change in SSA repair (bottom). (**c**) Western blot analysis of DNA2 knockdown. The average data from three independent experiments are shown, with the error bars representing standard deviation (n=3).

**Figure S6.** NSC-105808 reduces DSB repair by SSA. Cells were transfected with EGFP or I-SceI plasmids for 24 hrs and then cultured for an additional 48 hr before analysis by flow cytometry (top). Treatment with NSC-105808 was for 24 hrs prior to flow cytometry. Bar graph showing SSA efficiency in cells treated with NSC-105808 as compared to control cells treated with DMSO. The average data from three independent experiments are shown, with the error bars representing standard deviation (n=3, bottom).

**Figure S7:** Cell cycle distribution of U2OS cells upon treatment with NSC-105808. (**a**) U2OS cells were treated with DMSO or the indicated concentration of NSC-105808 for 24 hrs followed by a pulse of BrdU (10 µM) for 30 minutes and then analyzed by flow cytometry. (**b**) Representative cell cycle profiles are shown. (**c**) Quantitation of the G1 cell population. The results shown represent the mean ± SD of three independent experiments.

**Figure S8.** Analysis of DNA2 protein levels in cells treated with NSC-105808. U2OS (**a**), PANC-1 (**b**) and Hs578T (**c**) cells were treated with the indicated concentrations of NSC-105808 for 4 hours (right) or with a single concentration of NSC-105808 (0.3 μM for U2OS and Hs578T cells; 0.6 μM for PANC-1 cells) for 0, 4, 8, or 12 hrs (left). The results were quantified and are shown below each panel, with the error bars representing the mean ± SD of three independent experiments. No statistically significant difference in the DNA2 protein level was found in any of the cell lines.

**Figure S9.** (**a**) Western blot analysis of oncogenic Ras expression in indicated inducible cell lines. (**b**) MTT assay of cell proliferation in control and K-Ras-induced HPDE cells. Proliferation of indicated cell lines in response to a 96 hr treatment with the indicated concentrations of NSC-105808. Proliferation relative to cells treated with vehicle alone was determined by the MTT assay. Each value represents the mean ± SEM from three independent experiments. (**c**) Analysis of colony formation in control and K-Ras-induced HPDE cells treated with NSC-105808. Each value is relative to the untreated control group of control cells. The graph represents the mean ± SEM from three independent experiments. * p<0.05.
