# Supplementary material for: Inhibition of DNA2 nuclease as a therapeutic strategy targeting replication stress in cancer cells
Source: Oncogenesis. 2017 Apr 17;6(4):e319–. doi: 10.1038/oncsis.2017.15 (PMC5520492; doi:10.1038/oncsis.2017.15)
Supplement: Supplementary Material Methods [file oncsis201715x4.docx]

**Supplemental Material**

**Supplemental Methods**

***Cell proliferation assay.***

To determine the cell proliferation rate, 1000 cells/well were seeded in a 96-well plate in a total volume of 100 μl in triplicate in each experiment. On the following days, 20 μl of MTT substrate (2 mg/ml) was added to each well. After a 3-hr incubation, the culture medium was removed, and 100 μl of dimethyl sulfoxide was added. Plates were read at 490 nm and 650 nm (background) in a

microplate reader (Molecular Devices). After the subtraction of background, cell viability was calculated as fold change relative to control cells.

***Colony-forming assay***

DNA2 knockdown cells were seeded at low density; cells were then left for 2 weeks to allow colonies to form. Cells were stained with 2% methylene blue/50% ethanol, and colonies containing 50 or more cells were counted. To test the sensitivity of FANCD2-deficient cells to NSC-105808 and cisplatin, cells were seeded at 25 cells/well in 6-well plates. Cells were treated with cisplatin (0.3125 μM) together with or without NSC-105808 (0.016 μM). Colonies were fixed, stained, and scored ten days after treatment.

***Senescence assay***

For senescence assay cells were seeded in 6-well plates. After washing with PBS, cells were incubated with 1x fixation solution containing 2% formaldehyde and 0.2% glutaraldehyde in PBS. The cells were washed again and stained for 4 hr with 1 mg/ml X-gal, 40 mM citric acid/sodium phosphate (pH 6.0), 150 mM NaCl, and 2 mM MgCl_2_. The stain was removed, cells were rinsed with PBS, and positive staining was viewed and scored.

***Gene conversion and SSA Repair Assays***

The GFP expressing plasmid (pEGFP-C1) was used for transfection efficiency control. Cells were transfected with the pCBASce plasmid. Forty-eight hours after pCBASce transfection, flow cytometry was performed to detect GFP-positive cells using a FACScalibur apparatus with the CellQuest software (Becton Dickinson, San Jose, CA). NSC-105808 at indicated concentrations was added for 24 hrs before the analysis by flow cytometry. Prior to I-SceI transfection, cells were transfected twice with DNA2 siRNA at 24 hrs and 48 hrs in order to knockdown DNA2. Efficiency of HR and SSA was calculated by normalizing I-SceI-induced GFP expression with EGFP transfection-induced GFP expression. Each value presented at histograms is a fold change compared to the I-SceI-transfected control cells set to 1.

***Protein purification***

DNA2, DNA2 nuclease dead mutant (D277A), BLM, RPA, and EXO1 were purified as previously described ^14^.

***In vitro cell assay of K-Ras inducible HPDE cells***

MTT (Sigma; M5655) was used to evaluate the proliferation of cells. Briefly, 1000 doxycycline inducible HPDE cells were counted and seeded in a 96-well flat-bottomed plate, cells were incubated without and with doxycycline (10mg/ml) at 1:5000 ratio. NSC-105808 was added at 0, 0.125 µM, 0.25 µM, 0.5 µM and 1µM respectively. After 96 h, cells were incubated with MTT substrate (Sigma; 20 mg/ml) for 4 h, and the cultures were removed and replaced with dimethyl sulfoxide. The optical density was measured spectrophotometrically at 570 nm. The colony formation assay was performed by seeding 500 cells in six-well plates. 0.25 µM NSC-105808 was added to the culture medium, and the cells were compared with untreated control cells. Colonies were scored after 2 weeks. All experiments were repeated three times.

***Tumor Growth in Nude Mice***

Female athymic nu/nu mice (6–8 weeks old) were used for in vivo xenograft studies. Mice were quarantined for at least 1 week before experiments. All animal studies were conducted in compliance with protocols approved by the MDACC Institutional Animal Care and Use Committee. Exponentially growing Aspc1 (1 × 10^6^) was implanted subcutaneously at the flank of nude mice (left: control cells; right: DN2A knock down, n=5). Tumors were measured every 2 days by caliper and tumor volume was determined by using the formula [length/2] × [width2]. For immunohistochemistry, tumor tissue samples were fixed in 4% buffered paraformaldehyde and processed for histopathologic evaluation by paraffin embedding and antibody staining. Quantitative analysis of IHC for both phospho-CHK1(S345) and Ki67 (n=3) was performed by ImageJ.
